# A Novel Lytic *Salmonella* Phage Harboring an Unprecedented Tail-Protein Domain Combination Capable of Lysing Cross-Host-Transmitted *Salmonella* Strains

**DOI:** 10.3390/foods14162850

**Published:** 2025-08-17

**Authors:** Ling Zhang, Mingqiang Guo, Xiaoyu Ma, Wei Wang, Wanpeng Ma, Yifan Liu, Junxiang Wei, Zhanqiang Su

**Affiliations:** 1 College of Veterinary Medicine, Xinjiang Agricultural University, Urumqi 830052, China; zlinn6@outlook.com (L.Z.); 18160656593@163.com (Y.L.);; 2 Xinjiang Key Laboratory of Herbivore Drug Research and Creation, Xinjiang Agricultural University, Urumqi 830052, China

**Keywords:** *Salmonella*, lytic phage, tailspike protein, PFGE, biological control

## Abstract

The emergence of multidrug-resistant *Salmonella* poses a significant threat to global public health and food safety, necessitating the urgent search for new strategies to replace conventional antibiotics. Phages are viruses that can directly target bacteria and have garnered attention in recent years for their development as antibiotic alternatives. In this study, 4458 samples were collected from farms, supermarkets, and human feces, yielding 65 strains of *Salmonella*, which were serotyped using multiplex PCR. Subsequently, a lytic phage was isolated and identified using the dominant serotype of *Salmonella* as the host bacterium. We further explored the biological characteristics of this phage through host range, growth properties, and genomic analysis. Finally, we analyzed the potential of the phage to block the cross-host transmission of *Salmonella*, combining PFGE *Salmonella* classification, strain sources, and phage lytic phenotypes. The results showed that phage gmqsjt-1 could lyse 69.23% (45/65) of *Salmonella*, of which 75.56% (34/45) were resistant strains. The optimal multiplicity of infection (MOI) for gmqsjt-1 was 0.01, with a latent period of about 10 min, maintaining high activity within the temperature range of 30 to 60 °C and pH range of 2 to 13. No virulence or resistance genes were detected in the gmqsjt-1 genome, which carries two tail spike proteins (contain FAD binding_2 superfamily, the Tail spike TSP1/Gp66 N-terminal domain, and the Pectin lyase fold) and a holin–lysozyme–spanin lytic system. Phylogenetic classification indicates that phage gmqsjt-1 belongs to a new genus and species of an unnamed family within the class Caudoviricetes. PFGE classification results show a high genetic relationship among human, farm animal, and food source *Salmonella*, and the comprehensive lytic phenotype reveals that phage gmqsjt-1 can lyse *Salmonella* with high genetic correlation. These results suggest that this novel lytic *Salmonella* phage has the potential to inhibit cross-host transmission of *Salmonella*, making it a promising candidate for developing alternative agents to control *Salmonella* contamination sources (farms), thereby reducing the risk of human infection with *Salmonella* through ensuring food system safety.

## 1. Introduction

According to estimates from the World Health Organization in 2024, approximately 600 million people (one in every ten individuals) fall ill each year worldwide due to the consumption of contaminated food, resulting in 42,000 deaths. Unsafe food causes productivity and medical cost losses of up to USD 110 billion in low- and middle-income countries. https://www.who.int/news-room/fact-sheets/detail/food-safety (20 June 2025). *Salmonella* is one of the most common foodborne pathogens, causing over 90 million infections and more than 150,000 deaths each year [1]. *Salmonella enteritidis* (*S. enteritidis*)and *Salmonella typhimurium* (*S. typhimurium*) are the most common serovars causing foodborne disease outbreaks globally [2]. The United States Food and Drug Administration (FDA) and other agencies have confirmed four *S. typhimurium*/*enteritidis* outbreaks linked to melons from southwestern Indiana—occurring in 2012, 2020, 2022, and 2023—that were caused by environmental contamination in the growing area [3]. These incidents underscore the critical need to reduce *Salmonella* contamination in melon production regions. In Iran, during 2021–2022, *S. typhimurium* was the dominant serotype (82.5%) among diarrheal patients whose infections were potentially linked to poultry products and cross-contaminated foods [4]. Similarly, in China, 46.96% of foodborne salmonellosis cases reported between 2013 and 2022 were attributable to *S. typhimurium* and *S. enteritidis*, with fruits and meats (including their processed products) identified as the primary sources, underscoring the critical need for enhanced food-safety interventions [5]. Furthermore, fluoroquinolones and β-lactams are first-line drugs for human treatment of *Salmonella* infections, especially for systemic infections or in immunocompromised patients [6]. Antibiotics are also the primary means of treating *Salmonella* infections in animals [7,8]. However, the rapid evolution of bacterial drug resistance, which is driven by the overuse of antibiotics in clinical settings, the long-term application of antibiotics in animal feed, and the discharge of drug residues through wastewater, has led to increased treatment risks, higher medication costs, food safety crises, and others, posing significant public health challenges [9,10,11]. Consequently, researchers have developed a variety of alternative therapeutic approaches, such as phage therapy, antimicrobial peptide therapy, and the application of natural compounds, to decrease the reliance on traditional antibiotics [12,13,14].

Phages are now considered an ideal tool for food-microbiological control because of their exquisite specificity for target bacteria, negligible impact on eukaryotic cells and the commensal microbiota, capacity for autonomous replication, and environmentally benign profile. Their potential to prevent foodborne disease is therefore substantial. In 2006, the United States approved ListShield™, a Listeria monocytogenes-specific phage cocktail, as a surface decontaminant for ready-to-eat poultry [15]. Subsequently, phage-based preparations targeting *Salmonella*, *Escherichia coli*, and *Listeria* have been licensed as food additives in the United States, Canada, Israel, Brazil, and other jurisdictions, with applications spanning chicken, seafood, red meat, eggs, and dairy products [16]. Although most attention has focused on post-harvest processing, the pre-harvest health status of food animals largely determines the prevalence of foodborne pathogens. The United States Food Safety and Inspection Service (FSIS) addressed this priority by issuing Directive 7120.1, which authorizes pre-slaughter administration of phages to livestock and poultry [17]. Commercial phage formulations—including Bafasal^®^, INSPEKTOR^®^, and SalmoFree^®^—developed in Europe, Brazil, and Colombia, have demonstrated robust safety and prophylactic efficacy in both broiler and laying hens [18,19,20].

Nevertheless, current research remains largely confined to isolated nodes of the food-production continuum, leaving the potential of phages to interrupt the entire farm-to-fork-to-human transmission chain largely unexplored. Here, we analyzed 4458 samples collected between 2017 and 2023 from farm animals, retail meats, and human diarrheal stools, yielding 65 *Salmonella* isolates whose serotypes were determined. Predominant serovars were used to isolate and characterize lytic phages, whose biological properties and genomic features were subsequently elucidated. Finally, PFGE-based subtyping combined with lysis phenotyping was employed to evaluate the capacity of these phages to curtail *Salmonella* transmission across animal, food, and human reservoirs, thereby providing a theoretical framework for their deployment as biocontrol agents throughout the food-production chain.

## 2. Materials and Methods

### 2.1. Samples and Bacterial Strains

A total of 65 strains of *Salmonella*, which were collected from 4458 anal swabs, excrement, and retail meat samples from humans and different animals (dogs, bovine, sheep, pork, chickens, geese, and pigeons) in Xinjiang from 2017 to 2023 (Supplementary Tables S1 and S2) were included in this study. The drug resistance of the strain is known. The phages were isolated from sewage near the pigeon field.

### 2.2. Identification of Salmonella Serotypes by Multiplex PCR

Kim et al.’s method was used to identify common clinical serotypes of *Salmonella* [21]. Briefly, two multiplex PCR assays were designed based on the genetic loci of *S. typhimurium* LT2 and *S. typhimurium* CT18 to determine the serotype of *Salmonella*. Different serotypes correspond to different amplification patterns.

### 2.3. Phage Isolation and Purification

The methods previously used in our laboratory were used for the isolation and purification of phages [22]. In a nutshell, a mixture the filtered sewage and bacterial suspension was incorporated into LB medium with 0.5% agar, and that evenly coated onto LB solid media with 1.5% agar. After it was cultured at 37 °C for 10 h, clear plaque was selected for purification.

### 2.4. Transmission Electron Microscopy (TEM)

The experimental method used in this study was described in detail in our previous paper [22]. In summary, phages from purification suspension were dropped onto carbon-coated copper mesh grid and were incubated for 10 min at room temperature. Then they were added to 2% (*w/v*) phosphotungstic acid solution for staining. Phage morphology was observed at an accelerated voltage of 120 kv using TEM (Tecnai 12).

### 2.5. Host Rang of Phage

The same method in our previous paper was used [22]. Specifically, After the phage was mixed with the bacterial suspension, the mixture was added to LB soft agar (0.5%) and overlaid onto an LB agar plate (1.5%). The plates were then incubated at 37°C for 10 h, and phage plaques were observed. This test included 65 strains of *Salmonella* with from different sources and serotypes (Supplementary Table S2).

### 2.6. Growth Characteristics: The Optimal MOI and One-Step Experiments

According to the previous research methodology, some adjustments were made to the test in this study [22]. The ratio of phage/bacterial concentration was adjusted to 0.00001, 0.0001, 0.001, 0.01, 0.1, 1, and 10 (Supplementary Table S3), and mixtures were cultured at 37 °C with 180 r/min for 4 h. The double-layer plate method was used to determine phage titer. The test was repeated three times. The OMOI was the mixture ratio with the highest titer of phage.

As described previously with minor modifications, the one-step growth curve of phage was measured [22]. According to the ratio of OMOI, the mixture was cultured at 37 °C for 4 h. Excess phages were removed at 8000 r/min for 5 min. Precipitate resuspended in LB medium was cultured at 37 °C and phage titers were measured every 10 min using a double-layer plate method.

### 2.7. Sensitivity of Temperature and pH

The phage titer of the same concentration was measured every 10 min using the double plate method in water bath at different temperatures (30 °C, 40 °C, 50 °C, 60 °C, 70 °C, 80 °C).

The mixture of phage and bacteria with OMOI ratio was cultured in LB medium with different pH (2, 3, 4, 5, 6, 7, 8, 9, 10, 11, 12, 13, 14) concentrations at 37 °C for 6 h, and the phage titer at different PH was determined by double-layer plate method.

### 2.8. DNA Extraction and Complete Genome Sequencing

The phage genome was extracted using HiPure Viral DNA Kit D191 (Magen, Shanghai, China). Complete genome sequencing of phage was carried out using the Illumina HiSeq system (Illumina, San Diego, CA, USA, RRID: SCR_016386). The reads were QC and then assembled using velvet, gap filled with SSPACE and GapFiller [23,24,25,26,27].

### 2.9. Annotation of the Genome

Phage’s Sequence completeness, Lifestyle, Anti-CRISPR, Antimicrobial Resistance Gene and Virelent Factor were accessed via the PhageScope online website [28]. This study employed the Graphage framework, built upon a graph convolutional network (GCN), to predict the lifestyle of an unknown phage. First, the complete genome was converted into a gapped pattern graph (GPG) in which each node represents a trinucleotide (k = 3) and each edge encodes a gapped pattern between two k-mers separated by ≤2 nucleotides (d = 2). Concurrently, HMMER was used to scan the genome for 206 lysogeny-associated proteins, producing a 206-dimensional binary vector indicating presence or absence of each protein. The GPG together with the protein vector was then fed into a pre-trained Graphage model that uses a multi-layer GCN to generate low-dimensional embeddings and outputs the probability of the phage being temperate. A threshold of 0.5 was applied: probabilities above this value classified the phage as temperate, or otherwise as virulent [29]. The genome was annotated by PHASTEST 3.0 [30] and mapped by Proksee 1.2.0 [31].

### 2.10. Phylogenetic and Taxonomic Analysis

Eleven sequences, which exhibited an expectation value (E-Value) approaching 0 and a Percentage of Identity (Per. Ident) of over 90% when aligned with the reference sequence (PP530290), were selected from the NCBI database. The entire analysis was carried out by the VICTOR web service (https://victor.dsmz.de (9 August 2025))—an automated platform that integrates the authoritative taxonomic framework of the International Committee on Taxonomy of Viruses (ICTV) with the Genome-BLAST Distance Phylogeny (GBDP) algorithm [32], enabling high-precision phylogenetic analysis and classification of prokaryotic viruses through standardized distance calculations and optimized clustering [33]. The resulting intergenomic distances were used to infer a balanced minimum evolution tree with branch support via FASTME including SPR postprocessing for each of the formulas D0, D4, and D6, respectively. Branch support was inferred from 100 pseudo-bootstrap replicates each [34]. Taxon boundaries at the species, genus, and family levels were estimated with the OPTSIL program, the recommended clustering thresholds, and an F value (fraction of links required for cluster fusion) of 0.5 [33,35,36]. The results were visualized using Chiplot (https://www.chiplot.online/# (28 April 2025)) [37].

### 2.11. Prediction of Tail Spike Protein Conserved Domain

Phylogenetic analysis of tail spike protein sequence of phage gmqsjt-1 was performed using the Neighbor-Joining (N-J) method with 1000 bootstrap replicates in MEGA 11. Meanwhile, the protein sequence was submitted to the Conserved Domain Database (CDD) on the NCBI and the InterPro protein sequence resource classification library for domain prediction [38]. The results were visualized using Chiplot (https://www.chiplot.online/# (28 April 2025)) [37].

### 2.12. Pulsed Field Gel Electrophoresis of Salmonella

The DNA fingerprints of 38 strains of *Salmonella* were distinguished by the pulsed field gel electrophoresis (PFGE), which was used previously [39]. In brief, an agar-gel carrying chromosome DNA of *Salmonella* was digested by *Xhol* (TaKaRa Dalian, China) at 37 °C for 2 h. Separation of DNA fragments was conducted on a CHEF-Mapper (Bio-Rad Laboratories, Hercules, CA, USA) using a 6 V/cm with an angle of 120, at 14 °C for 19 h. After the gel has undergone staining (ethidium bromide of 1.0 mg/L) and decolorization, electrophoretic pattern is recorded by the GEL DOC XR System (Bio-Rad Laboratories, Hercules, CA, USA). The *Salmonella* serotype Braenderup H9812 (ATCC BAA-664) was selected to serve as the molecular weight marker. A similarity threshold of 80% was employed as the cutoff for determining genetic relatedness.

## 3. Result

### 3.1. Serotype Identification

**Figure 1:**
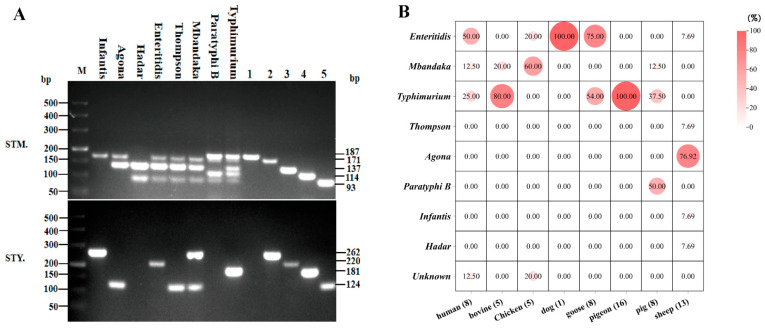
Serotype identification and analysis. (**A**) PCR amplification map of different serotypes of *Salmonella*. The upper figure (STM, the chromosomal regions of *S. enterica* serovars Typhimurium LT2) shows the amplification results of the specific primers for the STM group, which are STM1 (1: 187 bp), STM2 (2: 171 bp), STM3 (3: 137 bp), STM4 (4: 114 bp), and STM5 (5: 93 bp). The lower figure (STY, the chromosomal regions of *S. enterica* serovars Typhimurium CT18) shows the amplification results of the specific primers for the STY group, which are STY2 (2: 362 bp), STY3 (3: 220 bp), STM6 (4: 181 bp), and STY4 (5: 124 bp). M: DL500 DNA Marker. The second to the eighth lanes represent the amplification profiles of different serotypes. (**B**) Proportion of different animal *Salmonella* serotypes.

A total of 65 *Salmonella* strains were identified, representing eight serotypes: Infantis, Agona, Hadar, Enteritidis, Thompson, Mbandaka, Paratyphi B, and Typhimurium (Figure 1A). Among these, two could not be serotyped. Detailed results showed that *Salmonella* strains isolated from the feces of diarrheic patients and from sources related to bovine, dogs, geese, and pigeons were predominantly serotypes Enteritidis and Typhimurium. In contrast, strains associated with chickens, pigs, and sheep were mainly serotypes Mbandaka, Paratyphi B, and Agona (Figure 1B).

### 3.2. Isolation and Identification of Phages

**Figure 2:**
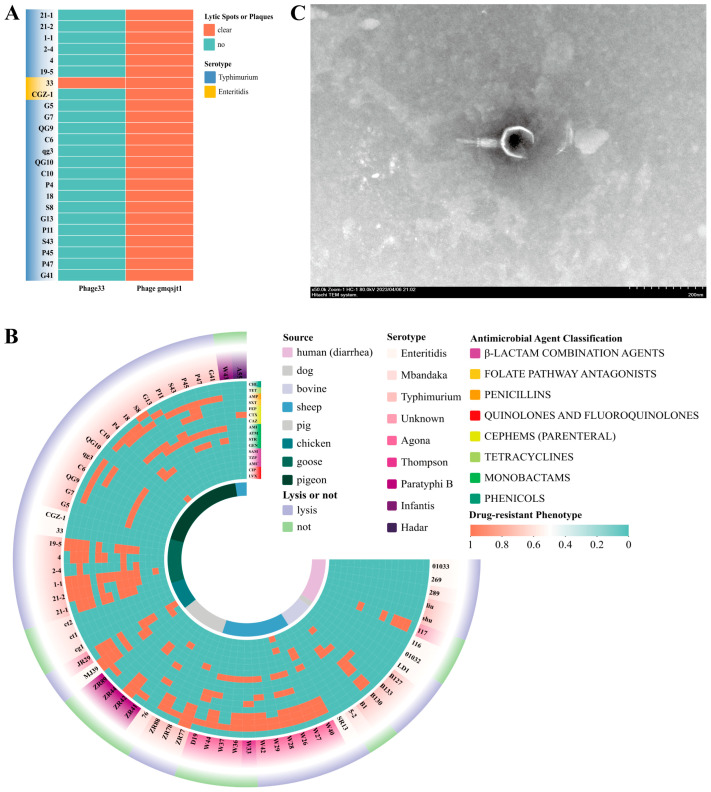
Phage host range. (**A**) The host range of two phages against 24 host strains. (**B**) The host range of phage gmqsjt-1 against 65 host strains. Transmission Electron Microscopy (TEM) image (the bar indicates 200 nm). (**C**) Phage gmqsjt-1 has a head of approximately 90 (±2) nm between opposite apices, and it is connected to a tail of about 130 (±2) nm.

Two *Salmonella* phages were isolated using 24 strains of *Salmonella* derived from pigeons and geese as host bacteria, one of which (Phage gmqsjt-1) exhibited the ability to lyse all 24 strains (Figure 2A). TEM showed that Phage gmqsjt-1 has a head of approximately 90 (±2) nm between opposite apices, and it is connected to a tail of about 130 (±2) nm (Figure 2C). Thus, Phage gmqsjt-1 belongs to the class Caudoviricetes [40]. The spot assay revealed sharply defined plaques of relatively uniform size (Supplementary Figure S1).

### 3.3. Phages Host Range

Phage gmqsjt-1 was used to lyse 65 strains of *Salmonella* in vitro (Figure 2B and Supplementary Table S2). The results demonstrated that it exhibited lytic activity against *Salmonella* strains of different origins and serotypes, with an overall lysis rate of 69.23% (45/65). Notably, the lysis rate against antibiotic-resistant strains was 73.91% (34/46). Of particular interest is that phage gmqsjt-1 lysed 62.50% (5/8) of the *Salmonella* strains isolated from human diarrheal fecal samples.

### 3.4. Growth Characteristics of Phage

**Figure 3:**
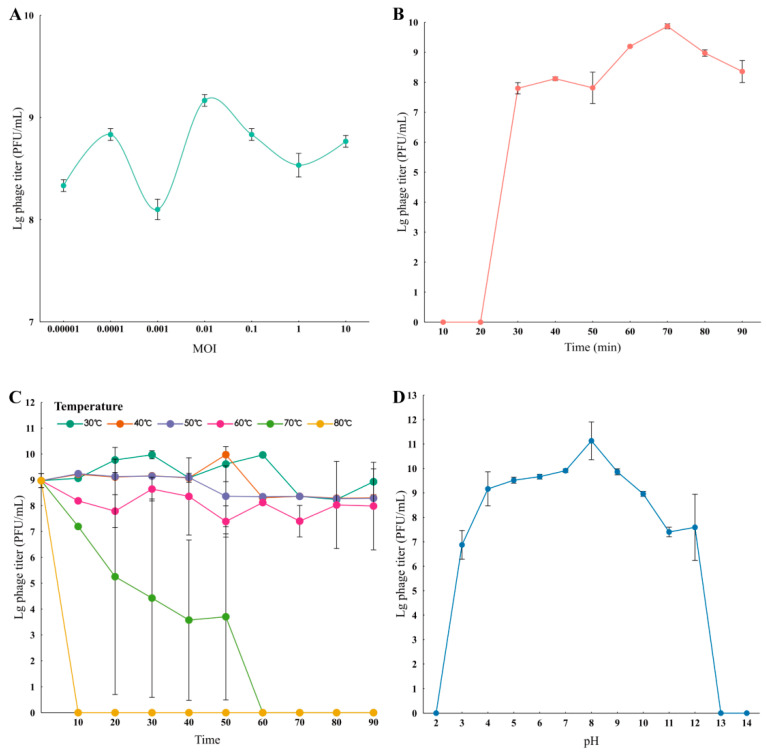
Growth characteristics of phage. (**A**) Optimal multiplicity of infection (OMOI) of phage. The highest titer of phage gmqsjt-1 was observed at a MOI of 0.01. (**B**) The one-step growth curve of phage. (**C**) Sensitivity of temperature. (**D**) Sensitivity of pH.

When the concentration ratio of phage gmqsjt-1 to host bacteria is 0.01, phage gmqsjt-1 exhibits OMOI (Figure 3A and Supplementary Table S3). As indicated by the one-step growth curve, the latent period of phage gmqsjt-1 was approximately 20 min, after which it entered the logarithmic growth phase within the subsequent 10 min. The phage titer reached its peak at 70 min (Figure 3B and Supplementary Table S4). The temperature sensitivity test revealed that phage gmqsjt-1 maintained relatively stable activity at temperatures of 30, 40, 50, and 60 °C. At 70 °C, the activity of phage gmqsjt-1 progressively declined over time and was completely lost within approximately 1 h. At 80 °C, the viability of phage gmqsjt-1 was limited to 10 min (Figure 3C and Supplementary Table S5). The pH sensitivity test demonstrated that phage gmqsjt-1 can survive across a broad pH range from 2 to 13. The highest phage titer was observed at pH 8 (Figure 3D and Supplementary Table S6).

### 3.5. Genome Analysis

**Figure 4:**
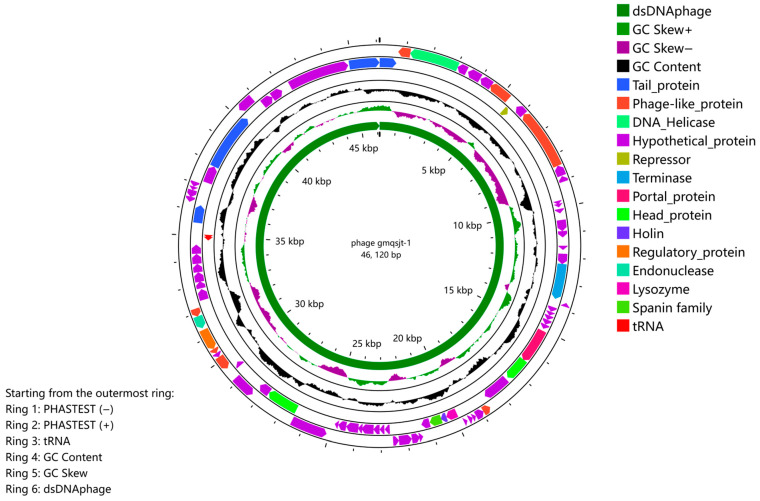
Genomic circle plot.

PhageScope analysis revealed that the genome of phage gmqsjt-1 possesses high integrity, with no predicted anti-CRISPR, antibiotic resistance, or virulence genes, indicating that it is a safe lytic phage. Phage gmqsjt-1 is a double-stranded DNA phage (dsDNAphage) with a genome size of 46,120 bp. A total of 79 coding sequences (CDSs) were annotated using PHASTEST 3.0, with lengths ranging from 96 to 2487 bp. Based on functional annotation, these CDSs were categorized into 13 classes, namely Tail_protein, Phage-like_protein, DNA_Helicase, Hypothetical_protein, Repressor, Terminase, Portal_protein, Head_protein, Holin, Regulatory_protein, Endonuclease, Lysozyme, and Spanin family. It should be highlighted that the tail structure of phage gmqsjt-1 is composed of four distinct elements: two tail spikes, one tail length tape-measure, and one major tail subunit phage. In addition, the phage carries the holin–lysozyme–spanin system (Figure 4 and Supplementary Table S7). The assembled sequence has obtained the accession (PP530290) of NCBI’s GenBank database.

### 3.6. Phylogeny and Classification

**Figure 5:**
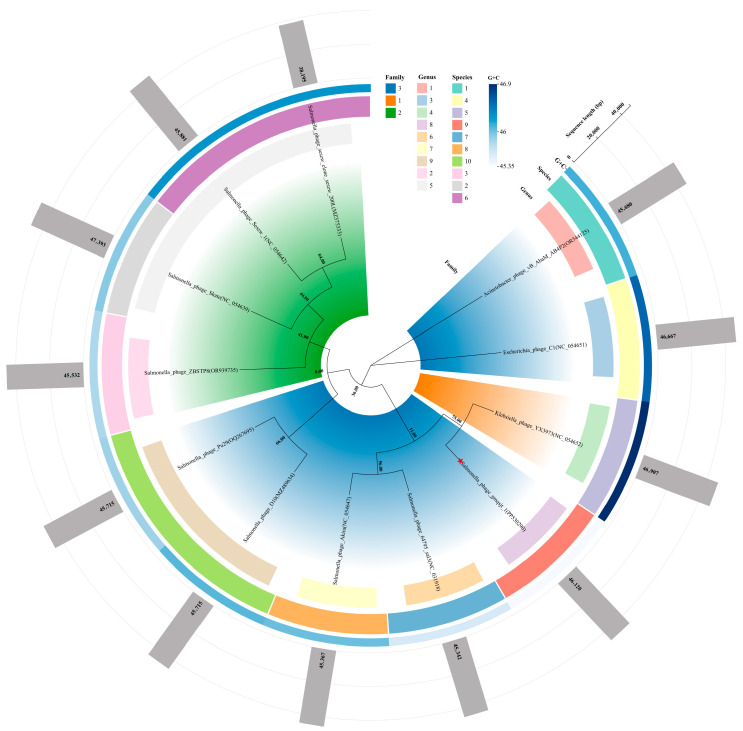
Phylogeny and classification. From the innermost ring outward: Ring 1, phylogenetic tree of the 12 genomes annotated at the family level; Ring 2, genus assignment of each genome; Ring 3, species assignment of each genome; Ring 4, G + C content of each genome; Ring 5, total amino-acid sequence size of each genome. The red five-pointed star, the phage gmqsjt-1 which is the main subject of this study.

Following 100 pseudo-bootstrap replicates, the mean branch support for distance formulas D0, D4, and D6 was 26%, 44%, and 29%, respectively. Consequently, the tree inferred with the D4 formula, which exhibited the highest average support, was selected for visualization and taxonomic interpretation. OPTSIL clustering, applied at the recommended thresholds (family ≥ 90%, genus ≥ 79%, species ≥ 70%), resolved the dataset into three families, nine genera, and ten species. The phage gmqsjt-1 grouped within the same family as *Salmonella* phages sal3 (NC_031918), Akira (NC_054647), D10 (MZ489634), Pu29 (OQ267695), C1 (NC_054651), and AB4P2 (OR544125). However, its genus and species-level assignments were distinct from all other genomes examined (Figure 5). Therefore, gmqsjt-1 represents a novel genus and species within an as-yet-unnamed family of the class Caudoviricetes.

### 3.7. Tail Spike Protein Domain

**Figure 6:**
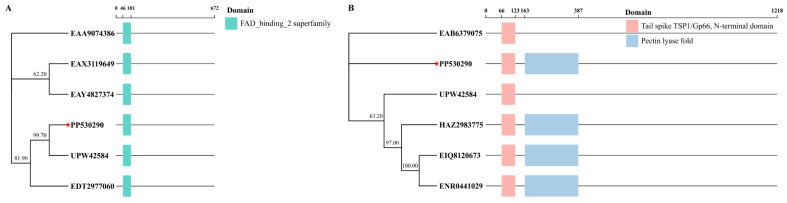
Prediction of tail spike protein domain. (**A**) Tail spike protein with 672 amino acids. (**B**) Tail spike protein with 1218 amino acids. The red five-pointed star, the phage gmqsjt-1 which is the main subject of this study.

Domain prediction was conducted for two tail spike proteins of phage gmqsjt-1. Tail spike protein with 672 amino acids was found to contain one FAD binding 2 superfamily, while tail spike protein with 1218 amino acids was identified to possess two distinct domains, namely Tail spike TSP1/Gp66, N-terminal domain, and Pectin lyase fold (Figure 6).

### 3.8. PFGE Analysis

**Figure 7:**
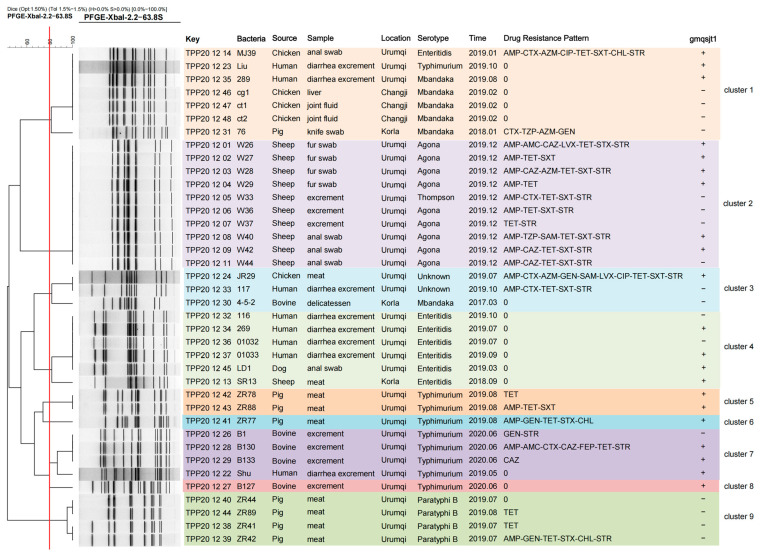
The dendrogram constructed from the pulsed-field gel electrophoresis patterns of the 38 *Salmonella* strains is presented. Different colors are used to indicate distinct PFGE clusters.

The screening of the 38 strains was centered on the background of human-derived *Salmonella* strains, primarily based on two criteria: year and region. According to the PFGE typing using the *XhoI* enzyme, the 38 strains were divided into nine clusters. Human-derived *Salmonella* strains were distributed in clusters 1, 3, 4, and 6. It is noteworthy that the human-derived *Salmonella* strains in clusters 1, 3, and 4 exhibited a similarity of nearly 100% to chicken-derived and dog-derived strains, respectively. It is noteworthy that the human-derived *Salmonella* strains in clusters 1, 2, and 4 exhibited a similarity of nearly 100% to chicken- and dog-derived strains. Additionally, the human-derived *Salmonella* strains in clusters 1, 2, 3, and 4 showed a similarity of over 80% to pig- and cattle-derived strains. These findings indicate a close genetic relationship between human- and animal-derived *Salmonella* strains. Considering the sample collection timeline, it is reasonable to infer that the *Salmonella* strains causing human diarrhea may be associated with chickens, dogs, pigs, and cattle. Taking into account the complexity of *Salmonella* strains derived from chickens, the bacteria present in chicken feces, internal organs, and raw meat may all potentially be transmitted to humans through certain pathways, leading to human diarrhea (Figure 7).

Further analysis of the relationship between phage lysis and PFGE typing reveals that phage gmqsjt-1 is capable of lysing *Salmonella* strains from human, chicken, and dog sources with a similarity of 100% in clusters 1 and 4. However, in cluster 3, phage gmqsjt-1 lysed *Salmonella* from chicken meat but failed to lyse the human-derived *Salmonella* with a similarity of 100%. A similar phenomenon was observed in cluster 2 (Figure 7).

## 4. Discussion

*Salmonella* are ranked by the WHO as the fourth leading etiological agent of diarrheal disease globally [41]. Non-typhoidal *Salmonella* (NTS) not only causes severe foodborne illnesses in humans but also infects poultry, livestock, and other animals, leading to significant economic losses in the livestock industry [42,43,44]. In this study, 93.65% (59/63) of the *Salmonella* isolates were identified as NTS, suggesting the widespread dissemination potential of NTS. Notably, *S. enteritidis* and *S. typhimurium* have consistently ranked as the top two serovars in the EU’s One Health zoonosis reports since 2018 [45]. In addition, they are also the most common serotypes causing human invasive NTS diseases through the food chain [46]. These two serotypes accounted for 63.49% (40/63) of *Salmonella* isolates with known serotypes in this study. The presence of PFGE clusters 4 and 7 further demonstrates that *Salmonella* transmission patterns in Xinjiang, China, are consistent with this epidemiological trend. However, it should be particularly noted that the *S*. Mbandaka from chicken and pig sources are clustered into the same PFGE cluster as human-derived. Currently, no reports of human infections with *Salmonella* enterica serovar Mbandaka have been documented in the Asian region. Dating back to 2020, Mbandaka has been consistently ranked within the top 20 serotypes of confirmed human salmonellosis cases in the European Union’s zoonoses reports [47]. This suggests that the potential threat of *S*. Mbandaka to humans in Asia may have been underestimated.

The emergence of multidrug-resistant *Salmonella* has significantly complicated clinical treatment, necessitating urgent development of novel prevention and control strategies [48]. Phages—viruses that specifically infect and lyse bacteria—exhibit high host specificity and have been successfully applied across multiple domains, including food industry applications, veterinary medicine, and human therapeutics [49,50,51]. The isolated phage gmqsjt-1 in this study represents a novel phage, which shows no genus-level affiliation with other *Salmonella* phages in the class *Caudoviricetes*, and has been confirmed as a new unnamed species through ANI analysis. This phage exhibited an MOI of 0.01 and demonstrated lytic activity against 69.23% (45/65) of the *Salmonella* isolates in this study, among which 73.91% (34/45) were drug-resistant strains. Notably, it also lysed *Salmonella* strains from eight different sources. The superior infectivity and lytic efficacy of phage gmqsjt-1 against *Salmonella* can be attributed to its dual tail spike proteins and a holin–lysozyme–spanin lysis system. The structural complex formed by the tail spike protein domains—TSP1/Gp66-N-terminal and pectin lyase fold—plays a dual role in the early infection phase, mediating both host recognition and pre-lytic functions, thereby significantly enhancing host adaptability and lysis efficiency [52,53]. Furthermore, the pectin lyase fold domain exhibits additional capacity to suppress host defense mechanisms [54]. In the holin–lysozyme–spanin system, holin forms holes in the cell membrane from the inside of the bacteria, releasing endolysin to degrade peptidoglycan, and then spanin destroys the outer membrane and eventually leads to cell disintegration [55,56]. The synergistic action of these two systems enables phage gmqsjt-1 to exhibit broad-spectrum lytic activity across diverse host strains.

Phage environmental stress tolerance is critical for cross-host and cross-domain applications. In this study, phage gmqsjt-1 maintained relatively stable activity after 90 min exposure to temperatures below 70 °C, demonstrated acid-base resistance, and effectively suppressed bacterial growth even at an ultra-low MOI of 1 × 10^−5^. Phage gmqsjt-1 demonstrates superior thermal tolerance compared to *Salmonella* phage SP154 (employed in milk and chicken processing) [57]. Furthermore, it achieves significantly lower MOI thresholds than poultry-farm-utilized *Salmonella* phage vB_SalP_LDW16 [58] while maintaining acid-base resistance profiles equivalent to food-ingredient-adapted *Salmonella* phages [59]. According to the performance of phage gmqsjt-1 in PFGE clusters 1, 3, 4, and 7, phage gmqsjt-1 not only has the potential to be applied to the environment such as meat products, clinical treatment, or aquaculture, or even extreme environments such as dairy products or sewage treatment, but also can reduce the potential risk of phage by reducing the dosage. In addition, the genome of phage gmqsjt-1 does not carry any virulence genes and drug resistance genes, which further indicates that phage gmqsjt-1 has a high safety potential in disinfection and treatment.

Pulsed-field gel electrophoresis (PFGE) is the “gold standard” for identifying the transmission routes and outbreak sources of pathogens [60]. In 2020, a large-scale outbreak of foodborne enteritis in Italy was monitored by PFGE and found to be associated with livestock production facilities located near cheese processing plants [61]. Another outbreak of salmonellosis in South Korea in 2021 was confirmed to be associated with eggs through PFGE [62]. In this study, PFGE clusters 1, 3, 4, and 7 confirmed the potential link between human-derived *Salmonella* infections and sources such as chicken swabs, chicken viscera, raw chicken, pig slaughter knives, dog fecal swabs, raw mutton, and cow feces. It is worth noting that strains from the same sheep farm and time period in cluster 2 exhibited a high degree of similarity in their antibiotic resistance phenotypes, indicating that horizontal gene transfer may have occurred during the spread of the strains. However, the phages were only able to lyse some strains in cluster 2, suggesting that the horizontal gene transfer (HGT) among the strains may have affected phage resistance genes (such as CRISPR spacers, restriction–modification systems, etc.), thereby influencing the phage lysis effect. However, this does not affect the PFGE typing [63,64]. In addition, the lysis effect of phages is also related to factors such as mutations in bacterial surface receptor molecules and lysogenic immunity [65,66]. Nevertheless, this study can still reflect through the correlation between phage lysis effects and PFGE tracing that phages gmqsjt-1 have the potential to cut off the possibility of foodborne disease outbreaks at their source of transmission (such as farms, pets themselves, or food).

## 5. Conclusions

In conclusion, the main serotypes of Salmonella prevalent in Xinjiang, China, are *S. enteritidis* and *S. typhimurium*, which are consistent with international reports. However, the harm caused by S. Mbandaka in the Asian region may have been underestimated. This study isolated a new genus and new species of phage, gmqsjt-1, from the class Caudoviricetes, which is an unnamed family. It not only has safety properties but also shows good tolerance to temperature and pH. The tail protein carried by phage gmqsjt-1 has a new domain combination and has the potential to block the cross-species transmission of *Salmonella*. Therefore, it is worth in-depth study of the lysis mechanism of this new type of phage.

## Data Availability

The original contributions presented in this study are included in the article/Supplementary Materials. Further inquiries can be directed to the corresponding author.

## Supplementary Materials

The following supporting information can be downloaded at https://www.mdpi.com/article/10.3390/foods14162850/s1; Table S1: Source information of strains; Table S2: *Salmonella* isolates used to examine the host range of phage; Table S3: The optimal multiplicity of infection (MOI); Table S4: One-step experiments; Table S5: Temperature sensitivity test; Table S6: PH sensitivity test; Figure S1: Phage plaque morphology.
