# Supplementary material for: A Novel Lytic Salmonella Phage Harboring an Unprecedented Tail-Protein Domain Combination Capable of Lysing Cross-Host-Transmitted Salmonella Strains
Source: Foods. 2025 Aug 17;14(16):2850. doi: 10.3390/foods14162850 (PMC12385820; doi:10.3390/foods14162850)

Supplementary Table S1 Source information of strains

	Healthy animal excrement/anal swab/fur swab/viscera/other swab							Retail meat (from the supermarket)				Diarrhea	Total
	Bovine	Sheep	Chicken	Pig	dog	goose	pigeon	Bovine	Sheep	Chicken	Pig	Human	
Total sample	1142	261	455	48	231	724	521	125	130	130	130	561	4458
Number of isolates	4	12	4	0	1	8	16	1	2	1	8	8	65
Separation rate (%)	0.35	4.60	0.88	0.00	0.43	1.10	3.07	1.60	0.77	0.77	6.15	1.43	1.46

Supplementary Table S2 *Salmonella* isolates used to examine the host range of phage

Bacteria	Source	Year	Sample	Serotype	Drug-resistant phenotype	gmqsjt1
01033	human (diarrhea)	2019	excrement	Enteritidis	/	+
269	human (diarrhea)	2019	excrement	Enteritidis	/	+
289	human (diarrhea)	2019	excrement	Mbandaka	/	+
Liu	human (diarrhea)	2019	excrement	Typhimurium	/	+
Shu	human (diarrhea)	2019	excrement	Typhimurium	/	+
117	human (diarrhea)	2019	excrement	Unknown	AMP-CTX-TET-SXT-STR	-
116	human (diarrhea)	2019	excrement	Enteritidis	/	-
01032	human (diarrhea)	2019	excrement	Enteritidis	/	-
LD1	dog	2019	anal swab	Enteritidis	/	+
B127	bovine	2020	excrement	Typhimurium	/	+
B133	bovine	2020	excrement	Typhimurium	CAZ	+
B130	bovine	2020	excrement	Typhimurium	AMP-AMC-CTX-CAZ-FEP-TET-STR	+
B1	bovine	2020	excrement	Typhimurium	GEN-STR	-

4-5-2	bovine	2017	delicatessen	Mbandaka	/	-
SR13	Sheep	2018	mutton	Enteritidis	/	+
W40	sheep	2019	anal swab	Agona	AMP-TZP-SAM-TET-SXT-STR	+
W27	sheep	2019	fur swab	Agona	AMP-TET-SXT	+
W26	sheep	2019	fur swab	Agona	AMP-AMC-CAZ-LVX-TET-SXT-STR	+
W28	sheep	2019	fur swab	Agona	AMP-CAZ-TET-SXT-STR	+
W29	sheep	2019	fur swab	Agona	AMP-TET	+
W42	sheep	2019	anal swab	Agona	AMP-CAZ-TET-SXT-STR	+
W33	sheep	2019	excrement	Thompson	AMP-CTX-TET-SXT-STR	-
W36	sheep	2019	excrement	Agona	AMP-TET-SXT-STR	-
W37	sheep	2019	excrement	Agona	TET-STR	-
W44	sheep	2019	fur swab	Agona	AMP-CAZ-TET-SXT-STR	-
D19	sheep	2019	fur swab	Agona	CTX-CAZ-TET	-
W43	Sheep	2019	excrement	Infantis	/	-
A55	Sheep	2019	mutton	Hadar	CTX	-
ZR77	pig	2019	raw meat	Typhimurium	AMP-GEN-TET-SXT-CHL	+
ZR78	pig	2019	raw meat	Typhimurium	TET	+
ZR88	pig	2019	raw meat	Typhimurium	AMP-TET-SXT	+
76	pig	2018	knife swab	Mbandaka	CTX-TZP-ATM-GEN	-
ZR41	pig	2019	raw meat	Paratyphi B	TET	-
ZR42	pig	2019	raw meat	Paratyphi B	AMP-GEN-TET-SXT-CHL-STR	-
ZR44	pig	2019	raw meat	Paratyphi B	/	-
ZR89	pig	2019	raw meat	Paratyphi B	TET	-
MJ39	chicken	2019	anal swab	Enteritidis	AMP-CTX-ATM-CIP-TET-SXT-CHL-STR	+
JR29	chicken	2019	joint fluid	Unknown	AMP-CTX-ATM-GEN-SAM-LVX-CIP-TET-SXT-STR	+
cg1	chicken	2019	liver	Mbandaka	/	-

ct1	chicken	2019	joint fluid	Mbandaka	/	-
ct2	chicken	2019	joint fluid	Mbandaka	/	-
21-1	goose	2021	anal swab	Typhimurium	AMP-CTX-CAZ-ATM-GEN-TET-SXT-STR	+
21-2	goose	2021	anal swab	Typhimurium	AMP-CTX-ATM-GEN-SAM-TET-SXT-CHL-STR	+
1-1	goose	2021	anal swab	Typhimurium	AMP-CTX-CAZ-ATM-GEN-SAM-AMI-CIP-TET-SXT-CHL-STR	+
2-4	goose	2021	anal swab	Typhimurium	AMP-SAM-STR	+
4	goose	2022	anal swab	Typhimurium	AMP-CTX-CAZ-TET-SXT-STR	+
19-5	goose	2021	anal swab	Typhimurium	APM-CTX-ATM-GEN-SAM-TET-SXT-CHL-STR	+
33	goose	2021	anal swab	Enteritidis	/	+
CGZ-1	goose	2021	anal swab	Enteritidis	/	+
G5	pigeon	2022	viscera	Typhimurium	STR	+
G7	pigeon	2022	viscera	Typhimurium	TET-STR	+
QG9	pigeon	2022	viscera	Typhimurium	TET-STR	+
C6	pigeon	2022	viscera	Typhimurium	TET-STR-SXT	+
qg3	pigeon	2022	viscera	Typhimurium	TET-STR-SXT	+
QG10	pigeon	2022	viscera	Typhimurium	/	+
C10	pigeon	2022	viscera	Typhimurium	TET	+
P4	pigeon	2022	anal swab	Typhimurium	CAZ-TET-STR-AMP-GEN-LVX	+
18	pigeon	2022	anal swab	Typhimurium	CAZ-TET-AMP-GEN	+
S8	pigeon	2022	viscera	Typhimurium	CAZ-AMP-CHL-AMI	+
G13	pigeon	2022	viscera	Typhimurium	TET-AMI	+
P11	pigeon	2023	viscera	Typhimurium	AMP-CAZ-AMI	+
S43	pigeon	2023	viscera	Typhimurium	AMP-FEP-STR-AMI	+
P45	pigeon	2023	viscera	Typhimurium	AMP-FEP-STR-SXT-AMI	+
P47	pigeon	2023	viscera	Typhimurium	AMP-AMI	+
G41	pigeon	2023	viscera	Typhimurium	AMP-STR-AMI	+

Note: AMC, Amoxicillin-clavulanate (20/10 µg/piece); TZP, Piperacillin-tazobactam (100/10 µg/piece); SXT, Sulfisoxazole (300 µg/piece); AMP, Ampicillin (10 µg/piece); SAM, Ampicillin-sulbactam (10/10 µg/piece); LVX, Levofloxacin (5 µg/piece); CAZ, Ceftazidime (30 µg/piece); CIP, Ciprofloxacin (5 µg/piece); TET, Tetracycline (30 µg/piece); CTX, Cefotaxime (30 µg/piece); GEN, Gentamicin (10 µg/piece); STR, Streptomycin (10 µg/piece); ATM, Aztreonam (30 µg/piece); FEP, Cefepime (30 µg/piece); AMI, Amikacin (30 µg/piece); CHL, Chloramphenicol (30 µg/piece).

Supplementary Table S3 The optimal multiplicity of infection (MOI)

Phage name	Bacterial Concentration ($CFU \cdot mL^{-1}$)	Phage Concentration ($PFU \cdot mL^{-1}$)	MOI	Phage Titer Repeat 1 ($PFU \cdot mL^{-1}$)	Phage Titer Repeat 2 ($PFU \cdot mL^{-1}$)	Phage Titer Repeat 3 ($PFU \cdot mL^{-1}$)
gmqsjt1	4.2×10^7	6.1×10^8	10	2.7×10^8	1.9×10^8	2.2×10^8
	4.2×10^7	6.1×10^7	1	6.4×10^8	7.8×10^8	6.8×10^8
	4.2×10^7	6.1×10^6	0.1	1.1×10^8	1.6×10^8	1.2×10^8
	4.2×10^7	6.1×10^5	0.01	1.21×10^9	1.44×10^9	1.46×10^9
	4.2×10^7	6.1×10^4	0.001	7.2×10^8	6.3×10^8	6.6×10^8
	4.2×10^7	6.1×10^3	0.0001	2.4×10^8	3.9×10^8	3.9×10^8
	4.2×10^7	6.1×10^2	0.00001	4.6×10^7	6.6×10^7	6.2×10^7

Supplementary Table S4 One-step experiments

Time (min)	phage titer ($PFU \cdot mL^{-1}$)		
	Repeat 1	Repeat 2	Repeat 3
10	0	0	0
20	0	0	0
30	5.7×10^7	1.01×10^8	4.3×10^7
40	1.24×10^8	1.18×10^8	1.54×10^8
50	1.27×10^8	1.62×10^7	1.34×10^8

60	1.69×10^9	1.49×10^9	1.53×10^9
70	5.9×10^9	8.4×10^9	7.9×10^9
80	1.24×10^9	8.6×10^8	7.8×10^8
90	2.8×10^8	0.9×10^8	4.7×10^8

Supplementary Table S5 Temperature sensitivity test

Time (min)		0	10	20	30	40	50	60	70	80	90
Temperature (°C)		phage titer (PFU·mL^{-1})									
	Repeat 1	4.6×10^8	9.4×10^8	1.44×10^{10}	1.31×10^{10}	7.6×10^8	6.7×10^9	8.9×10^9	1.94×10^8	1.65×10^8	2.24×10^8
30	Repeat 2	1.23×10^9	1.31×10^9	8.4×10^9	9.6×10^9	1.37×10^9	9.4×10^9	9.6×10^9	2.46×10^8	1.87×10^8	1.69×10^9
	Repeat 3	1.43×10^9	1.23×10^9	1.68×10^9	6.4×10^9	1.62×10^9	1.06×10^{10}	9.1×10^9	2.53×10^8	1.67×10^8	1.59×10^8
	Repeat 1	4.6×10^8	1.51×10^9	1.57×10^9	1.55×10^9	1.06×10^9	1.24×10^{10}	1.87×10^8	2.14×10^8	2.02×10^8	2.44×10^8
40	Repeat 2	1.23×10^9	1.73×10^9	1.16×10^9	1.36×10^9	1.37×10^9	8.6×10^9	2.47×10^8	2.32×10^8	1.76×10^8	1.73×10^8
	Repeat 3	1.43×10^9	1.65×10^9	1.14×10^9	1.41×10^9	1.14×10^9	7.8×10^9	1.75×10^8	2.47×10^8	2.07×10^8	1.92×10^8
	Repeat 1	4.6×10^8	1.64×10^9	1.46×10^9	1.16×10^9	1.37×10^9	1.02×10^8	1.87×10^8	2.17×10^8	1.76×10^8	1.87×10^8
50	Repeat 2	1.23×10^9	1.72×10^9	1.12×10^9	1.38×10^9	1.18×10^9	1.17×10^9	2.39×10^8	2.34×10^8	2.14×10^8	2.06×10^8
	Repeat 3	1.43×10^9	1.83×10^9	1.47×10^9	1.69×10^9	1.17×10^9	1.05×10^9	2.46×10^8	2.36×10^8	1.74×10^8	1.86×10^8
	Repeat 1	4.6×10^8	1.53×10^8	1.42×10^8	9.4×10^8	9.2×10^9	1.04×10^8	1.24×10^8	1.26×10^8	1.14×10^8	8.9×10^8
60	Repeat 2	1.23×10^9	1.64×10^8	1.13×10^8	6.8×10^8	1.27×10^8	1.17×10^8	1.43×10^8	1.08×10^8	9.4×10^9	1.07×10^7
	Repeat 3	1.43×10^9	1.45×10^8	1.44×10^8	1.32×10^9	1.02×10^8	1.21×10^8	1.29×10^8	1.17×10^8	1.13×10^8	9.5×10^8
	Repeat 1	4.6×10^8	1.62×10^7	8.4×10^7	4.1×10^6	2.2×10^5	4.1×10^5	0	0	0	0
70	Repeat 2	1.23×10^9	1.47×10^7	0	4.6×10^6	2.4×10^5	0	0	0	0	0
	Repeat 3	1.43×10^9	1.62×10^7	6.8×10^7	0	0	3.1×10^5	0	0	0	0
	Repeat 1	4.6×10^8	0	0	0	0	0	0	0	0	0
80	Repeat 2	1.23×10^9	0	0	0	0	0	0	0	0	0
	Repeat 3	1.43×10^9	0	0	0	0	0	0	0	0	0

Supplementary Table S6 PH sensitivity test

PH	phage titer (PFU·mL^{-1})		
	Repeat 1	Repeat 2	Repeat 3
2	0	0	0
3	7.0×10^5	3.0×10^5	2.0×10^5
4	4.9×10^7	9.2×10^8	6.9×10^7
5	2.6×10^8	4.5×10^8	3.1×10^5
6	5.8×10^8	4.6×10^8	3.7×10^8
7	6.7×10^9	8.9×10^9	9.0×10^9
8	9.7×10^{10}	2.42×10^9	8.4×10^{10}
9	5.7×10^8	6.9×10^8	9.9×10^8
10	7.8×10^7	1.2×10^6	8.1×10^7
11	2.3×10^7	4.1×10^7	1.7×10^7
12	2.0×10^6	1.0×10^8	3.0×10^6
13	0	0	0
14	0	0	0

Supplementary Figure S1 Phage plaque morphology

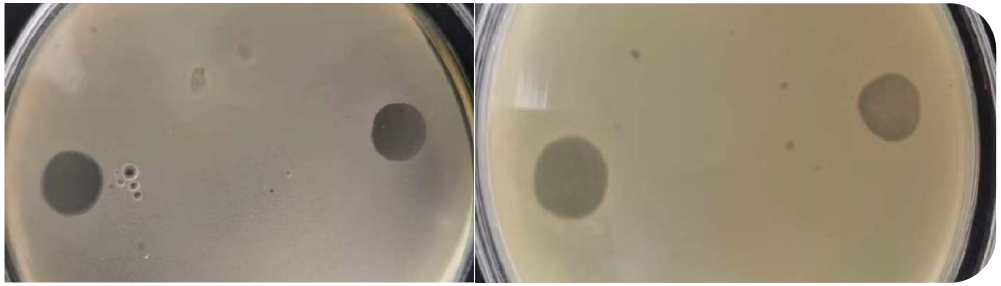

Supplement: Supplementary file 1 [file foods-14-02850-s001.zip › supplementary data.pdf]
